# Sleep Quality in Patients with Epilepsy: Differences in Anxiety, Depression, and Clinical Characteristics

**DOI:** 10.3390/medicina62020403

**Published:** 2026-02-19

**Authors:** Silvija Bartašiūnaitė, Dovydas Burkojus, Agnė Šmigelskytė, Giedrė Jurkevičienė, Giedrė Gelžinienė

**Affiliations:** 1 Department of Neurology, Lithuanian University of Health Sciences, A. Mickevičiaus Str. 9, LT-44307 Kaunas, Lithuania; 2 Neurology Department, Hospital of Lithuanian University of Health Sciences Kauno Klinikos, Affiliated Member of the European Reference Network EpiCARE, LT-50161 Kaunas, Lithuania

**Keywords:** epilepsy, sleep quality, insomnia, excessive daytime sleepiness, depression, anxiety

## Abstract

*Background and Objectives*: People with epilepsy frequently complain of poor sleep quality, excessive daytime sleepiness (EDS), and insomnia. Therefore, this study aimed to evaluate differences in anxiety and depression symptoms, as well as clinical characteristics, across groups defined by sleep quality in patients with epilepsy. *Materials and Methods:* Seventy-eight adults with epilepsy were assessed using standardized questionnaires for sleep quality (Pittsburgh Sleep Quality Index, PSQI), daytime sleepiness (Epworth Sleepiness Scale, ESS), insomnia severity (Insomnia Severity Index, ISI), and psychiatric symptoms (PHQ-9, GAD-7, and HADS). Demographic data (age and sex), seizure frequency and characteristics, use of antiepileptic drugs (AEDs), and EEG findings were collected. Patients were divided into groups based on sleep quality scores, and comparisons were made regarding anxiety, depression, and selected clinical variables. Associations were analyzed using t-tests, chi-squared tests, and Spearman correlation coefficients. *Results:* Poor sleep quality (PSQI > 5) was present in 70.9% of patients and was significantly associated with insomnia, daytime sleepiness, depression, and anxiety symptoms (*p* < 0.001 for all comparisons). Patients who had experienced generalized tonic–clonic seizures (GTCS) in the past year had significantly worse sleep quality compared to those without GTCS (*p* = 0.025). Clinical insomnia (ISI ≥ 15) was observed in 23.1% of cases and was significantly associated with the presence of seizures (*p* = 0.015). EDS was present in 19% of cases and was associated with depressive symptoms (*p* = 0.019). A higher concentration of levetiracetam was associated with better sleep quality, whereas a higher concentration of lamotrigine was associated with worse sleep quality (*p* = 0.024 for both). EEG abnormalities, seizure frequency, and duration of epilepsy were not associated with sleep quality. *Conclusions:* Poor sleep quality was reported in 70% of the study patients and was associated with increased insomnia severity, EDS, and psychiatric comorbidities. People with EDS were more likely to have higher levels of depression and anxiety. Patients who experienced GTCS within the past year were significantly more likely to report poor sleep quality. Insomnia was associated with older age and female sex. Seizure-free patients had less insomnia. Nevertheless, no associations were found between sleep evaluation scores and other demographic or clinical epilepsy characteristics.

## 1. Introduction

Epilepsy is a chronic neurological condition characterized by recurrent seizures. It is one of the most common neurological conditions, affecting about 50 million people globally [1]. Epilepsy is a complex disorder affecting multiple domains of health, including sleep. Sleep and epilepsy have a bidirectional relationship: epilepsy can negatively impact the quality of sleep, while seizures can be exacerbated by disturbed sleep [2,3,4].

Common sleep issues among patients with epilepsy include insomnia and excessive daytime sleepiness (EDS), and they often report an overall poor quality of sleep. These issues are more common in patients with epilepsy than in the general population [2,5,6]. Sleep disorders might not only compromise patients’ general health but also the management of seizures, as sleep deprivation is a well-known risk factor for seizure recurrence. In addition, among patients with epilepsy, sleep disturbances are strongly linked to symptoms of anxiety and depression. According to various studies, 9.5–26% of patients with epilepsy suffer from depression, and 22.8–39.4% of patients experience anxiety [7]. Psychotropic drugs prescribed for depression and anxiety might cause various drug interactions, increase the risk of side effects, and lead to poorer adherence to a medication regimen, which further contributes to fatigue and emotional instability, closely related to insufficient sleep [8,9]. This relationship further complicates the management of epileptic seizures [3,6,7,8,10].

Antiepileptic drugs (AEDs) may influence sleep architecture and quality. Some AEDs, including higher doses of levetiracetam (LEV) and phenobarbital, may have sedative properties and cause daytime sleepiness [2,11]. AED polytherapy is linked to major disruptions in sleep [5,12]. These findings emphasize the need for customized treatment plans to maximize seizure control and maintain sleep quality.

Epileptic seizures and sleep have common neurophysiological features, involving thalamo-cortical circuits, NREM oscillations, and brain plasticity mechanisms [13]. Electroencephalogram (EEG) changes during sleep may be associated with sleep disturbances in patients with epilepsy. In non-rapid eye movement (NREM) sleep stage three, sleep spindles, K-complexes, and slow waves may facilitate interictal epileptiform discharges (IED) and seizure progression [3,14,15,16]. Focal IED may occur more frequently during slow-wave sleep and sometimes spread bilaterally or become generalized [1,4,17]. Increased IEDs during NREM sleep are thought to result from the development of neuronal synchrony within brainstem–thalamocortical networks during this sleep phase [18]. The presence of epileptiform discharges leads to repeated arousals and sleep fragmentation, eventually contributing to excessive daytime sleepiness.

Considering these complex links, this study aims to explore the relationships between sleep quality, anxiety, depression symptoms, sleep EEG changes, and AED concentrations in patients with epilepsy. Our secondary aims include (1) assessing demographic and clinical characteristics, (2) examining associations between sleep quality and emotional disturbances, (3) analyzing the presence of epileptiform discharges in EEG and their relations to sleep evaluation scores, (4) exploring links to AED levels, and (5) investigating variations based on epilepsy clinical characteristics. The evaluation of different characteristics of epilepsy, along with mood disorders and anxiety, and their associations with sleep problems is important, as they can negatively affect seizure control and mood. Therefore, the improvement of sleep practices can become an additional non-pharmacological treatment method for epilepsy and common comorbid psychiatric disorders.

## 2. Materials and Methods

This cross-sectional observational study included 78 adult patients diagnosed with epilepsy, recruited from the Department of Neurology at the Hospital of Lithuanian University of Health Sciences Kauno Clinics, Kaunas, Lithuania, from February to May 2025. Inclusion criteria were age ≥18 years, confirmed diagnosis of epilepsy based on ILAE criteria, and the ability to provide informed consent and complete self-reported questionnaires. Patients with major cognitive impairment, active psychosis, or other neurological or psychiatric conditions that could independently influence sleep quality were excluded. All other patients who agreed to participate were included consecutively, without further selection based on clinical features, EEG availability, or other characteristics.

Clinical data were collected through a review of medical records. Variables included age, sex, onset age and duration of epilepsy, seizure type (focal vs. generalized or bilateral TCS), seizure frequency, treatment type (monotherapy vs. polytherapy), AED concentration, and history of generalized tonic–clonic seizures (GTCS) during the last year and over the patient’s lifetime. Blood samples for the evaluation of concentrations of AED in most of the patients were taken before the usual morning dose; fasting status was not evaluated. Seizure freedom was defined as no clinical seizures in the past 12 months. Routine and sleep EEG recordings were evaluated by neurologists as part of standard clinical practice. Most patients underwent daytime sleep EEG rather than overnight recordings due to limited availability. Findings were categorized and compared between groups based on sleep quality (PSQI) and other clinical variables.

Sleep quality was assessed using the Pittsburgh Sleep Quality Index (PSQI), with scores > 5 indicating poor sleep. EDS was evaluated using the Epworth Sleepiness Scale (ESS), with a score ≥10 indicating pathological sleepiness. Severity of insomnia was measured using the Insomnia Severity Index (ISI), with a score ≥ 15 classified as clinical insomnia. Psychiatric symptoms were assessed using the Patient Health Questionnaire-9 (PHQ-9) for depressive symptoms; the Generalized Anxiety Disorder-7 (GAD-7) for anxiety; the Hospital Anxiety and Depression Scale (HADS), including separate anxiety (HADS-A) and depression (HADS-D) subscales.

Data were analyzed with IBM SPSS v. 29.0. An independent samples t-test was used for the comparison of continuous variables, and chi-squared tests were used for categorical variables. Spearman correlation analysis was used to evaluate the relationships between ordinal variables. Statistical significance was defined as *p* < 0.05. Given the exploratory nature of the study, statistical analyses were performed to assess associations and group differences, and *p*-values should be interpreted descriptively.

The Kaunas Regional Bioethics Committee approved the conduct of the study (BE-2-14). The patients provided their written informed consent to participate in this study.

## 3. Results

### 3.1. Demographic and Clinical Characteristics

The study included 78 patients with epilepsy (63.3% female), with a mean age of 37.6 (SD ± 15.4) years. The mean age at epilepsy onset was 22.9 (SD ± 18.2) years, and the mean duration of epilepsy was 14.7 (SD ± 10.4) years. Most patients had focal epilepsy (66.7%). One-third were seizure-free in the past year, while nearly a quarter (23.1%) experienced frequent seizures (more than three per month). GTCS were highly prevalent—more than a third experienced them in the past year. Epileptiform discharges were found in 21 of 66 individuals (31.8%) who underwent routine EEG and in 13 of 21 patients (61.9%) who underwent sleep EEG. Detailed clinical and demographic characteristics are presented in Table 1.

The majority of patients had poor sleep quality (PSQI mean = 7.99 ± 4.03). Excessive daytime sleepiness (ESS > 9) was present in 19% of patients. Insomnia symptoms (ISI mean = 9.64 ± 6.83) were reported by 23.1%, meeting criteria for moderate to severe insomnia. Depression symptoms (PHQ-9) averaged 7.53 ± 6.06; moderate or worse symptoms were present in 35% of participants. Anxiety symptoms were present in 45.5% of patients according to the GAD-7, with 22.1% reporting mild, 13.0% moderate, and 10.4% severe symptoms. Based on HADS-A scores, 24.7% of participants scored ≥ 8, indicating clinically significant anxiety. Full sleep and psychiatric profiles are provided in Table 2.

### 3.2. Sleep Quality and Clinical Correlations

Based on the PSQI, 70.9% of patients had poor sleep quality (PSQI > 5) (Table 3). Compared with good sleepers (PSQI < 5, *n* = 23), they did not differ in age, sex, epilepsy type and duration, seizure frequency, or treatment type (*p* > 0.05). However, poor sleepers had significantly higher scores for insomnia (ISI: 11.9 ± 6.6 vs. 4.2 ± 3.4; *p* < 0.001), daytime sleepiness (ESS: 6.9 ± 4.1 vs. 4.0 ± 2.7; *p* < 0.001), depression (PHQ-9: 9.4 ± 6.2 vs. 3.3 ± 2.7; *p* < 0.001), and anxiety scales (GAD-7: 8.0 ± 6.1 vs. 2.8 ± 3.3; HADS-A: 6.7 ± 5.2 vs. 3.1 ± 2.6; both *p* < 0.001). Moderate to severe depressive symptoms were present in nearly half (48.1%) of poor sleepers compared to only 4.5% of good sleepers (*p* = 0.001). Additionally, poor sleep quality was more frequent in patients who had GTCS in the past year (42.6% vs. 13.0%; *p* = 0.025).

The occurrence of epileptiform activity on sleep EEG did not differ among individuals with poor sleep quality (69.2% vs. 50.0%).

### 3.3. Excessive Daytime Sleepiness and Clinical Correlations

In this study, 15 participants (19%) were identified with EDS, defined by an ESS score >9. When comparing demographic factors (age, sex) and epilepsy-related clinical characteristics (epilepsy type and duration, seizure frequency, and treatment), no statistically significant differences were found between individuals with and without EDS (*p* > 0.05).

However, patients with EDS reported significantly poorer sleep quality (PSQI: 9.9 ± 3.5 vs. 7.5 ± 4.1; *p* = 0.028) and showed higher depressive symptoms (PHQ-9: 11.2 ± 6.2 vs. 6.7 ± 5.7; *p* = 0.019) (Table 4). Although insomnia and anxiety levels were elevated in EDS patients, they did not achieve statistical significance.

### 3.4. Insomnia and Clinical Correlates

Clinical insomnia was present in 23.1% of patients with epilepsy and was associated with older age and female sex. The prevalence of insomnia differed across patient groups according to seizure frequency (*p* = 0.015). Patients in remission more frequently reported no insomnia, although no linear trend was observed (linear-by-linear association *p* = 0.762), indicating that insomnia did not increase proportionally with seizure frequency (Table 5). Patients with insomnia reported poorer sleep quality, more daytime sleepiness, and significantly higher levels of depression and anxiety. No differences were found between groups according to EEG findings or GTCS presence.

### 3.5. Associations of Blood Concentrations of Antiepileptic Drugs (AED) with Sleep and Psychological Symptoms

The concentration of levetiracetam (LEV) in blood was significantly associated with better sleep quality, diminished insomnia, and reduced anxiety and depression symptoms (Table 6). However, lamotrigine (LTG) concentration correlated with worse outcomes across sleep and mood domains. Blood concentrations of other AEDs, such as valproic acid, carbamazepine, and topiramate, showed no significant association. Due to the very small number of users, we did not calculate correlations for oxcarbazepine, pregabalin, ethosuximide, and phenobarbital.

## 4. Discussion

The findings of our study support the hypothesis that poor sleep quality is highly prevalent among individuals with epilepsy. Results show that poor sleep quality is strongly associated with increased insomnia and EDS. Poor sleep quality in our research was strongly associated with elevated depression and anxiety symptoms, consistent with prior research indicating a bidirectional link between sleep and mood disorders [19,20,21,22]. Insomnia was strongly associated with higher depression and anxiety scores across all scales. Similar associations have been reported by Im et al. [19] and Planas-Ballvé et al. [21], emphasizing that insomnia in epilepsy is closely tied to psychological distress, often independent of seizure burden. In our study, we did not find correlations between any clinical epilepsy characteristics and insomnia scores. These findings support the need for routine mood and sleep screening in people with epilepsy.

Patients who experienced GTCS within the past year were more likely to report poor sleep quality. This corresponds with prior studies showing that the presence of recent GTCS, but not necessarily their frequency, can negatively influence subjective sleep quality [21]. The association between GTCS and sleep difficulties can be explained through physiological, psychological, and treatment-related mechanisms. First, GTCS can cause functional changes in neuronal networks, especially in thalamocortical circuits, which are necessary for preserving normal sleep architecture. This can lead to reduced sleep efficiency, more awakenings, and shorter REM/deep sleep stages [23,24]. Second, GTCS—especially those experienced at night—can cause great psychological discomfort and raise anxiety about nighttime seizures. Patients may have trouble falling asleep, and secondary insomnia may develop even after seizures are controlled [21,25]. Treatment might be changed after seizures (increased AED doses and/or adding another AED), which in turn can also cause drowsiness or compromise the quality of sleep [11,26]. These complex factors indicate that the impact of GTCS on sleep is multi-dimensional and involves both direct neurophysiological effects and indirect psychological and pharmacological aspects.

Epileptiform discharges in sleep EEG were more common among poor sleepers in our study, but the difference was not statistically significant. Still, data from some other studies suggest that subtle changes in sleep architecture may underlie subjective sleep complaints in epilepsy. A meta-analysis by Lehner et al. found that patients with idiopathic generalized epilepsy exhibited measurable changes in sleep architecture, including reduced sleep efficiency and increased REM latency [23]. A separate meta-analysis by Yeh et al. further showed that patients with focal epilepsy had significantly reduced REM sleep compared to controls [24]. Using intracranial EEG and simultaneous polysomnography, Peter-Derex et al. revealed a possible relationship between interictal and ictal discharges and an increase in arousals and awakenings in people with epilepsy [27]. These findings indicate that epileptiform discharges may disrupt sleep physiology in various epilepsy subtypes, potentially going undetected by routine clinical EEG but still having a negative influence on sleep quality. Therefore, overnight recording of sleep EEG could be useful, but this type of evaluation requires additional human resources and is not routinely available in general clinical settings.

Contrary to some earlier studies [3,21], we did not find significant demographic or clinical associations with sleep evaluation results, except for insomnia. According to our data, 23.1% of patients with epilepsy had clinical insomnia, which was associated with older age, female sex, and seizure frequency. As people age, the prevalence of insomnia rises because of gradual alterations in the circadian clock and sleep homeostasis processes, as well as the development of chronic illnesses that alter sleep patterns [28]. We found that patients who are free of seizures had significantly less insomnia, but no linear link with seizure frequency was established. These findings may be attributed to the complex and reciprocal relationship between insomnia and seizure frequency. Quigg et al. [25] also observed that the persistence of seizures, even at low frequency, was associated with more severe insomnia and reduced quality of life. Furthermore, other studies demonstrate that seizure frequency and type can disrupt sleep architecture and exacerbate subjective sleep complaints [12,21]. Moreover, Alenizi et al. noted that patients with monthly seizures were 2.5 times more likely to experience poor sleep compared to those with annual seizures, suggesting an association between active epilepsy and sleep disturbance [29]. Further, sleep disturbance can in turn exacerbate seizures, although this is not uniform in all cases. It is imperative to discern sleep–wake patterns of individual patients, for changes in these patterns may be associated with seizures [30]. Idiopathic generalized epilepsy, such as juvenile myoclonic epilepsy and epilepsy with generalized tonic–clonic seizures alone, is especially prone to sleep deprivation with seizures occurring after awakening [31]. Moreover, inconsistencies in sleep schedule may reduce sleep duration, which in turn negatively impacts seizure control in focal patients with epilepsy [32]. In our study, we did not evaluate differences between insomnia and poor seizure control separately in generalized and focal epilepsy patient groups. However, our study includes patients from a single tertiary center and revealed overall an alarming association between sleep deprivation and poor seizure control, indicating that sleep disturbances, such as insomnia, must be taken into account upon patient visits.

GTCS and interictal EEG abnormalities are often considered potential contributors to sleep disruption in epilepsy [21]. In our study, the presence of recent GTCS was linked to worse total sleep quality. We did not find significant differences in EEG changes or GTCS history between patients with and without clinical insomnia. This suggests that seizure type, severity, or underlying electrophysiological abnormalities may not be the only factors contributing to insomnia in epilepsy. Consistent with other studies, this might suggest that insomnia is more closely associated with mental and behavioral issues, like depression, anxiety, and worry about future seizures [19,21]. On the other hand, standard clinical EEG, typically performed during wakefulness or a short period of sleep, may not detect subtle disruptions in sleep architecture, such as reduced slow-wave sleep or REM fragmentation, which are better revealed by full-night polysomnography [23,24].

EDS was found in 19% of patients with epilepsy, and these individuals reported significantly poorer sleep quality and more depressive symptoms. Multiple studies report diminished sleep quality and elevated EDS among patients with epilepsy [3,18], and the relationship between EDS and poor sleep quality carries a clear bidirectional relationship [19]. Notably, in our study, 66.7% of those with EDS had moderate to severe depression, compared to 27.8% in the non-EDS group. This association between EDS and depression symptoms could be due to neurochemical and behavioral links between these two conditions. For instance, atypical depression frequently involves increased daytime sleepiness linked to serotonin and dopamine system disruptions [33]. Moreover, the mental exhaustion and reduced motivation common in depression could amplify the feeling of sleepiness experienced by patients with epilepsy [19]. While the severity of insomnia and anxiety was elevated in the EDS group, these did not reach statistical significance. The result is likely to be explained by several factors. Firstly, when identifying differences in psychiatric symptoms, the EDS group had a comparatively small sample size, which limits statistical power. Secondly, since both insomnia-related hyperarousal and hypersomnia-related daytime fatigue can impact mood regulation, the symptom overlap between the two conditions may mask clear group differences [34]. Third, regardless of sleep disturbance profiles, the expression of psychiatric symptoms in epilepsy is very diverse, with significant individual variations in the severity of anxiety and depression [35].

When evaluating AED use in our study, we found that LEV concentration was associated with better sleep quality, less insomnia, and reduced symptoms of anxiety and depression among patients with epilepsy. These findings are consistent with recent polysomnographic research [36] showing that LEV, especially in monotherapy, maintains overall sleep continuity without significantly impairing total sleep time, efficiency, REM, or arousal levels, despite slight shifts in sleep stage distribution. Similarly, a literature review [11] showed that LEV tends to have a neutral or slightly positive impact on sleep. However, a large database study found that LEV had the highest psychiatric and behavioral side effects and intolerability rates among 18 AEDs studied, including irritability, depressive mood, and aggression [37]. This contradiction might be possible because of population heterogeneity, different co-existing health conditions, variations in drug dosage, and the specific therapeutic setting of LEV use (alone versus combined therapy). Due to these well-known possible negative effects on mood, LEV is usually avoided in patients with psychiatric symptoms, which might also explain the lower depression and anxiety scores in our study. Our findings support the possible positive effect of LEV on sleep within a rather emotionally stable population.

In our study group, LTG plasma levels were associated with worse sleep quality, increased levels of insomnia, and more evident symptoms of anxiety and depression. Several studies analyzing LTG’s influence on sleep found that this drug may increase the risk of insomnia and is associated with poorer sleep quality [11,26,38]. Nevertheless, LTG is generally well-tolerated, with a lower incidence of mood disturbances compared to other AEDs [37]. It is possible that LTG was chosen over other AEDs to prevent mood-related adverse effects for individuals who exhibited anxiety or depression symptoms prior to initiating treatment. Since we do not have data about mood evaluation before AEDs were started, our results regarding this aspect should be interpreted with caution.

No significant correlations were found between valproic acid concentrations and sleep or psychiatric scores. Previous studies have suggested that valproic acid generally has a neutral or even protective effect on sleep architecture, with minimal impact on REM or slow-wave sleep stages [11]. In our study, no reliable conclusions could be drawn for other less frequently used (oxcarbazepine, pregabalin, ethosuximide, and phenobarbital) antiepileptic drugs because of the small number of users.

Several methodological limitations should be considered when interpreting the results of this study. We have included a heterogeneous group of patients with epilepsy with different epilepsy types (focal and generalized), varying seizure frequencies, and diverse medication regimens; however, this does not reflect the real-world population of patients with epilepsy. On the other hand, the relatively small cohort (*n* = 78), recruited from a single tertiary care center, may not be representative of the broader epilepsy population. The study’s cross-sectional design does not allow us to establish causal relations. Self-report questionnaires have limited reliability and are vulnerable to biases due to their subjective nature. Future multicenter studies with more homogeneous cohorts are needed to build upon these findings and provide more reliable results.

## 5. Conclusions

Poor sleep quality was reported in 70% of the study patients and was associated with increased insomnia severity, EDS, and psychiatric comorbidities. People with EDS were more likely to have higher levels of depression and anxiety. Patients who experienced GTCS within the past year were significantly more likely to report poor sleep quality. Insomnia was associated with older age and female sex. Seizure-free patients had less insomnia. Nevertheless, no associations were found between sleep evaluation scores and other demographic or clinical epilepsy characteristics. 

These findings highlight the need for comprehensive epilepsy care that considers mental and sleep health in addition to seizure control. Larger cohorts and thorough sleep assessments, like overnight EEG and polysomnography, should be used in future studies to further investigate these relationships.

## Figures and Tables

**Table 1:** Clinical and demographic characteristics of the study population (*n* = 78).

Characteristic	Value
Age, mean ± SD, years	37.6 ± 15.4
Sex, *n* (%)	
Female	51 (65.4%)
Male	27 (34.6%)
Age at onset, years, mean ± SD	22.9 ± 18.17
Newly diagnosed	6 (7.7%)
Epilepsy duration, years, mean ± SD	14.7 ± 10.4
Type of epilepsy, *n* (%)	
Focal	52 (66.7%)
Generalized	26 (33.3%)
Seizure frequency (past 12 months), *n* (%)	
Seizure freedom	25 (32.05%)
<1 a month	19 (24.36%)
1–3 times a month	16 (20.51%)
>3 times a month	18 (23.08%)
Predominant seizure type, *n* (%)	
Focal	22 (28.20%)
Generalized or focal to bilateral tonic–clonic seizures	56 (71.80%)
Number of seizures in the last 12 months, mean ± SD	48.20 ± 94.96
Vagal nerve stimulator, *n* (%)	7 (8.9%)
Treatment type, *n* (%)	
Monotherapy	44 (57.9%)
Polytherapy	32 (42.1%)
Epileptiform discharges in routine EEG, *n* (%)	17 (25.8%)
Epileptiform discharges in sleep EEG	13 (62.0%)
GTCS present during lifetime	53 (68.0%)
GTCS during the last 12 months	26 (35.9%)
MRI changes:	
Normal	34 (43.6%)
Abnormal	26 (33.3%)
Not available/not performed	18 (23.1%)
Individual AED prescribed	
Levetiracetam, *n* (%)	31 (40.8%)
Concentration, µg/mL, mean (SD)	14.4 ± 9.6
Lamotrigine, *n* (%)	24 (31.6%)
Concentration, µg/mL, mean (SD)	8.9 ± 5.8
Valproic acid, *n* (%)	24 (31.6%)
Concentration, µg/mL, mean (SD)	57.3 ± 19.1
Carbamazepine, *n* (%)	11 (14.5%)
Concentration, µg/mL, mean (SD)	8.1 ± 3.0
Topiramate, *n* (%)	7 (9.2%)
Concentration, mean (SD)	8.2 ± 2.7
Others *, *n* (%)	9 (11.7%)

GTCS: generalized tonic–clonic seizures, SD: standard deviation, AED: antiepileptic drugs. * Clonazepam, oxcarbazepine, pregabalin, ethosuximide, and phenobarbital were prescribed individually to less than 5% of patients.

**Table 2:** Results of sleep questionnaires and mood evaluation.

Scale/Subscale	Values	Scale/Subscale	Values
PSQI, mean ± SD	7.99 ± 4.03	GAD-7, mean ± SD	6.45 ± 5.89
Good sleep (0–5)	23 (29.1%)	Minimal, *n* (%)	42 (54.54%)
Poor sleep (>5)	56 (70.9%)	Mild, *n* (%)	17 (22.08%)
ESS, mean ± SD	5.99 ± 3.96	Moderate, *n* (%)	10 (12.99%)
Normal, *n* (%)	64 (81.0%)	Severe, *n* (%)	8 (10.39%)
Pathologic excessive sleepiness (>9 points), *n* (%)	15 (19.0%)	HADS-A (anxiety), mean ± SD	5.58 ± 4.86
ISI, mean ± SD	9.64 ± 6.83	Minimal, *n* (%)	58 (75.33%)
Absence of insomnia, *n* (%)	37 (47.44%)	Mild, *n* (%)	6 (7.79%)
Subthreshold insomnia, *n* (%)	23 (29.49%)	Moderate, *n* (%)	8 (10.39%)
Moderate insomnia, *n* (%)	13 (16.67%)	Severe, *n* (%)	5 (6.49%)
Severe insomnia, *n* (%)	5 (6.41%)	HADS-D (depression), mean ± SD	4.13 ± 3.67
PHQ-9, mean ± SD	7.53 ± 6.06	Minimal, *n* (%)	63 (81.82%)
Minimal, *n* (%)	33 (42.86%)	Mild, *n* (%)	7 (9.09%)
Mild, *n* (%)	17 (22.08%)	Moderate, *n* (%)	7 (9.09%)
Moderate, *n* (%)	17 (22.08%)	Severe, *n* (%)	0
Moderately severe, *n* (%)	7 (9.09%)		
Severe, *n* (%)	3 (3.89%)		

PSQI: Pittsburgh Sleep Quality Index, ISI: Insomnia Severity Index, ESS: Epworth Sleepiness Scale, GAD-7: Generalized Anxiety Disorder-7 Scale, HADS: Hospital Anxiety and Depression Scale, PHQ-9: Patient Health Questionnaire, SD: standard deviation.

**Table 3:** Clinical characteristics of patients with epilepsy with and without poor sleep quality.

Characteristic		PSQI < 5	PSQI ≥ 5	*p*
Age, mean ± SD		36.0 ± 13.7	38.4 ± 16.3	0.497
Sex	Male, *n* (%)	5 (33.3%)	22 (34.9%)	0.907
	Female, *n* (%)	10 (66.7%)	41 (65.1%)	
Type of epilepsy	Focal, *n* (%)	19 (82.6%)	33 (60.0%)	0.095
	Generalized, *n* (%)	4 (17.4%)	22 (40.0%)	
Epilepsy duration, years, mean ± SD		13.7 ± 10.8	15.1 ± 10.5	0.585
Epilepsy onset, years, mean ± SD		22.3 ± 17.2	23.3 ± 18.8	0.822
Seizure frequency (past 12 months)	Seizure freedom, *n* (%)	11 (47.8%)	14 (25.0%)	0.240
	<once a month, *n* (%)	5 (21.7%)	14 (25.5%)	
	1–3 times a month, *n* (%)	4 (17.4%)	12 (21.8%)	
	>3 times a month, *n* (%)	3 (13.0%)	15 (27.3%)	
Treatment type	Monotherapy, *n* (%)	11 (50.0%)	21 (38.9%)	0.526
	Polytherapy, *n* (%)	11 (50.0%)	33 (61.1%)	
ISI	Total, mean ± SD	4.2 ± 3.4	11.9 ± 6.6	< 0.001
	No insomnia (0–4 points), *n* (%)	21 (95.5%)	38 (69.1%)	0.030
	Clinical insomnia (>15 points)	1 (4.5%)	17 (30.9%)	
ESS	Total, mean ± SD	4.0 ± 2.7	6.9 ± 4.1	< 0.001
	Normal, *n* (%)	22 (95.7%)	41 (74.5%)	0.066
	EDS, *n* (%)	1 (4.3%)	14 (25.5%)	
PHQ-9	Total, mean ± SD	3.3 ± 2.7	9.4 ± 6.2	< 0.001
	Minimal–mild, *n* (%)	21 (95.5%)	28 (51.9%)	< 0.001
	Moderate, *n* (%)	1 (4.5%)	16 (29.6%)	
	Severe, *n* (%)	0 (0.0%)	10 (18.5%)	
GAD-7	Mean ± SD	2.8 ± 3.3	8.0 ± 6.1	< 0.001
	Minimal and mild symptoms, *n* (%)	21 (95.5%)	37 (68.5%)	0.027
	Moderate and severe symptoms, *n* (%)	1 (4.5%)	17 (31.5%)	
HADS-A (anxiety)	Mean ± SD	3.1 ± 2.6	6.7 ± 5.2	< 0.001
	Normal–borderline symptoms	22 (100.0%)	41 (75.9%)	0.028
	Clinical symptoms	0	13 (24.1%)	
HADS-D (depression)	Mean ± SD	2.2 ± 1.9	5.0 ± 3.9	< 0.001
	Normal–borderline symptoms	22 (100.0%)	47 (87.0%)	0.182
	Clinical symptoms	0	7 (13.0%)	
GTCS present during lifetime	Yes, *n* (%)	13 (56.5%)	40 (74.1%)	0.210
	No, *n* (%)	10 (43.5%)	14 (25.9%)	
GTCS during the last 12 months	Yes, *n* (%)	3 (13.0%)	23 (42.6%)	0.025
	No, *n* (%)	20 (87.0%)	31 (57.4%)	
Sleep EEG	Epileptiform discharges	4 (50.0%)	9 (69.2%)	0.370
	Non-specific changes	3 (37.5%)	4 (30.8%)	

PSQI: Pittsburgh Sleep Quality Index, ISI: Insomnia Severity Index, ESS: Epworth Sleepiness Scale, GAD-7: Generalized Anxiety Disorder-7 Scale, HADS: Hospital Anxiety and Depression Scale, PHQ-9: Patient Health Questionnaire, GTCS: generalized tonic–clonic seizures, SD: standard deviation, EEG: electroencephalography. Statistical significance was set at *p* < 0.05.

**Table 4:** Clinical characteristics of patients with epilepsy with and without excessive daytime sleepiness.

Characteristic		Normal	EDS	*p*
Age, mean ± SD		37.8 ± 15.9	37.1 ± 14.2	0.854
Sex	Male, *n* (%)	22 (36.7%)	5 (27.8%)	0.487
	Female, *n* (%)	38 (63.3%)	13 (72.2%)	
Type of epilepsy	Focal, *n* (%)	43 (68.3%)	9 (60.0%)	0.761
	Generalized, *n* (%)	20 (31.7%)	6 (40.0%)	
Epilepsy duration, mean ± SD		14.0 ± 10.3	17.8 ± 11.4	0.251
Epilepsy onset, mean ± SD		23.9 ± 18.2	19.3 ± 18.7	0.400
Seizure frequency (past 12 months)	Seizure freedom, *n* (%)	24 (38.1%)	1 (6.7%)	0.115
	<once a month, *n* (%)	14 (22.2%)	5 (33.3%)	
	1–3 times a month, *n* (%)	11 (17.5%)	5 (33.3%)	
	>3 times a month, *n* (%)	14 (22.2%)	4 (26.7%)	
Treatment type	Monotherapy, *n* (%)	26 (42.6%)	6 (40.0%)	1.000
	Polytherapy, *n* (%)	35 (57.4%)	9 (60.0%)	
Insomnia (ISI)	Total, mean ± SD	9.1 ± 6.7	12.2 ± 7.1	0.146
	No insomnia (0–14 points), *n* (%)	49 (79.0%)	10 (66.7%)	0.499
	Clinical insomnia (>15 points)	13 (21.0%)	5 (33.3%)	
PSQI	Mean ± SD	7.5 ± 4.1	9.9 ± 3.5	0.028
	PSQI < 5, *n* (%)	22 (34.9%)	1 (6.7%)	0.066
	PSQI ≥ 5, *n* (%)	41 (65.1%)	14 (93.3%)	
PHQ-9	Mean ± SD	6.7 ± 5.7	11.2 ± 6.2	0.019
	Minimal–mild, *n* (%)	44 (72.1%)	5 (33.3%)	0.018
	Moderate, *n* (%)	11 (18.0%)	6 (40.0%)	
	Severe, *n* (%)	6 (9.8%)	4 (26.7%)	
GAD-7	Mean ± SD	6.0 ± 5.8	8.7 ± 6.2	0.129
	Minimal and mild symptoms, *n* (%)	48 (78.7%)	10 (66.7%)	0.521
	Moderate and severe symptoms, *n* (%)	13 (21.3%)	5 (33.3%)	
HADS-A (anxiety)	Mean ± SD	5.2 ± 4.7	7.3 ± 5.4	0.182
	Normal–borderline symptoms	52 (85.2%)	11 (73.3%)	0.475
	Clinical symptoms	9 (14.8%)	4 (26.7%)	
HADS-D (depression)	Mean ± SD	3.8 ± 3.6	5.6 ± 3.5	0.100
	Normal–borderline symptoms	56 (91.8%)	13 (86.7%)	0.906
	Clinical symptoms	5 (8.2%)	2 (13.3%)	
GTCS present during lifetime	Yes, *n* (%)	42 (66.7%)	11 (78.6%)	0.582
	No, *n* (%)	21 (33.3%)	3 (21.4%)	
GTCS during the last 12 months	Yes, *n* (%)	19 (30.2%)	7 (50.0%)	0.268
	No, *n* (%)	44 (69.8%)	7 (50.0%)	
Sleep EEG	Epileptiform discharges	11 (61.1%)	2 (66.7%)	0.914
	Non-specific changes	6 (33.3%)	1 (33.3%)	

PSQI: Pittsburgh Sleep Quality Index, ISI: Insomnia Severity Index, ESS: Epworth Sleepiness Scale, GAD-7: Generalized Anxiety Disorder-7 Scale, HADS: Hospital Anxiety and Depression Scale, PHQ-9: Patient Health Questionnaire, GTCS: generalized tonic–clonic seizures, EEG: electroencephalography, SD: standard deviation. Statistical significance was set at *p* < 0.05.

**Table 5:** Clinical characteristics of patients with epilepsy with and without insomnia.

Characteristic		No Insomnia	Clinical Insomnia	*p*
Age, mean ± SD		35.2 ± 14.9	46.3 ± 15.2	0.011
Sex	Male, *n* (%)	25 (42.4%)	2 (11.1%)	0.015
	Female, *n* (%)	34 (57.6%)	16 (88.9%)	
Type of epilepsy	Focal, *n* (%)	38 (64.4%)	14 (77.8%)	0.440
	Generalized, *n* (%)	21 (35.6%)	4 (22.2%)	
Epilepsy duration, mean ± SD		14.3 ± 10.9	16.2 ± 9.6	0.467
Epilepsy onset, mean ± SD		20.9 ± 18.0	30.1 ± 18.5	0.073
Seizure frequency (past 12 months)	Seizure freedom, *n* (%)	22 (37.3%)	3 (16.7%)	0.015
	<once a month, *n* (%)	9 (15.3%)	9 (50.0%)	
	1–3 times a month, *n* (%)	12 (20.3%)	4 (22.2%)	
	>3 times a month, *n* (%)	16 (27.1%)	2 (11.1%)	
Treatment type	Monotherapy, *n* (%)	28 (49.1%)	4 (22.2%)	0.082
	Polytherapy, *n* (%)	29 (50.9%)	14 (77.8%)	
PSQI	Mean ± SD	6.8 ± 3.2	12.2 ± 3.6	0.0001
	PSQI < 5, *n* (%)	21 (35.6%)	1 (5.6%)	0.030
	PSQI ≥ 5, *n* (%)	38 (64.4%)	17 (94.4%)	
ESS	Mean ± SD	5.5 ± 3.6	8.1 ± 4.6	0.037
	Normal, *n* (%)	49 (83.1%)	13 (72.2%)	0.499
	EDS, *n* (%)	10 (16.9%)	5 (27.8%)	
PHQ-9	Mean ± SD	6.4 ± 5.4	11.9 ± 6.4	0.004
	Minimal–mild, *n* (%)	44 (74.6%)	5 (29.4%)	0.002
	Moderate, *n* (%)	10 (16.9%)	7 (41.2%)	
	Severe, *n* (%)	5 (8.5%)	5 (29.4%)	
GAD-7	Mean ± SD	5.1 ± 5.1	11.4 ± 6.1	0.001
	Minimal and mild symptoms, *n* (%)	50 (84.7%)	8 (47.1%)	0.004
	Moderate and severe symptoms, *n* (%)	9 (15.3%)	9 (52.9%)	
HADS-A (anxiety)	Mean ± SD	4.7 ± 4.2	9.0 ± 5.6	0.008
	Normal–borderline symptoms	52 (88.1%)	11 (64.7%)	0.058
	Clinical symptoms	7 (11.9%)	6 (35.3%)	
HADS-D (depression)	Mean ± SD	3.6 ± 3.3	6.2 ± 4.2	0.031
	Normal–borderline symptoms	55 (93.2%)	14 (82.4%)	0.374
	Clinical symptoms	4 (6.8%)	3 (17.6%)	
GTCS present during lifetime	Yes, *n* (%)	41 (70.7%)	11 (61.1%)	0.636
	No, *n* (%)	17 (29.3%)	7 (38.9%)	
GTCS during the last 12 months	Yes, *n* (%)	16 (27.6%)	9 (50.0%)	0.139
	No, *n* (%)	42 (72.4%)	9 (50.0%)	
Sleep EEG	Epileptiform discharges	10 (55.6%)	3 (100.0%)	0.341
	Non-specific changes	7 (38.9%)	0	

PSQI: Pittsburgh Sleep Quality Index, ISI: Insomnia Severity Index, ESS: Epworth Sleepiness Scale, GAD-7: Generalized Anxiety Disorder-7 Scale, HADS: Hospital Anxiety and Depression Scale, PHQ-9: Patient Health Questionnaire, GTCS: generalized tonic–clonic seizures, EEG: electroencephalography, SD: standard deviation. Statistical significance was set at *p* < 0.05.

**Table 6:** Correlation between blood concentrations of antiepileptic drugs and scores on PSQI, ESS, ISI, HADS, GAD-7, and PHQ-9 scales.

Drug	PSQI	ESS	ISI	HADS-A	HADS-D	GAD-7	PHQ-9
Levetiracetam	−0.418 (0.024)	−0.179 (0.353)	−0.418 (0.024)	−0.584 (0.001)	−0.288 (0.138)	−0.610 (0.001)	−0.408 (0.031)
Valproic acid	−0.236 (0.267)	0.016 (0.940)	0.039 (0.858)	−0.200 (0.361)	−0.372 (0.081)	−0.211 (0.333)	−0.339 (0.114)
Lamotrigine	0.458 (0.024)	0.105 (0.625)	0.538 (0.007)	0.535 (0.008)	0.491 (0.017)	0.449 (0.032)	0.356 (0.095)
Topiramate	0.126 (0.788)	0.536 (0.215)	0.324 (0.478)	0.000 (1.000)	0.613 (0.144)	0.126 (0.788)	−0.090 (0.848)
Carbamazepine	−0.051 (0.883)	−0.196 (0.563)	0.032 (0.926)	0.068 (0.841)	0.209 (0.538)	−0.123 (0.718)	0.229 (0.499)

PSQI: Pittsburgh Sleep Quality Index, ISI: Insomnia Severity Index, ESS: Epworth Sleepiness Scale, GAD-7: Generalized Anxiety Disorder-7 Scale, HADS: Hospital Anxiety and Depression Scale, PHQ-9: Patient Health Questionnaire. Statistical significance was set at *p* < 0.05.

## Data Availability

Data sharing is not applicable to this article.

## Abbreviations

The following abbreviations are used in this manuscript:

AED	Anti-seizure Medication
EEG	Electroencephalogram
ESS	Epworth Sleepiness Scale
GAD-7	Generalized Anxiety Disorder-7 Scale
